# Editorial for the Special Issue on Micromachines for Non-Newtonian Microfluidics

**DOI:** 10.3390/mi13060906

**Published:** 2022-06-08

**Authors:** Lanju Mei, Shizhi Qian

**Affiliations:** 1 Department of Engineering and Aviation Sciences, University of Maryland Eastern Shore, Princess Anne, MD 21853, USA; 2 Department of Mechanical and Aerospace Engineering, Old Dominion University, Norfolk, VA 23529, USA; sqian@odu.edu

Microfluidics has seen a remarkable growth over the past few decades, with its extensive applications in engineering, medicine, biology, chemistry, etc. Many of these real applications of microfluidics involve the handling of complex fluids such as whole blood, protein solutions, and polymeric solutions which exhibit non-Newtonian characteristics—specifically, viscoelasticity. The elasticity of the non-Newtonian fluids induces intriguing phenomena such as elastic instability and turbulence even at extremely low Reynolds numbers. This is the consequence of the nonlinear nature of the rheological constitutive equations. The nonlinear characteristics of non-Newtonian fluids can dramatically change the flow dynamics, and is useful to enhance mixing at the microscale. Electrokinetics in the context of non-Newtonian fluids are also of significant importance, with their potential applications in micromixing enhancement and bio-particles manipulation and separation. This Special Issue comprises 14 original contributions.

Regarding the instability of non-Newtonian fluids, Zhang et al. [1] demonstrate the viscoelastic fluid instability in an asymmetric nozzle–square microchannel. It is found that the critical Weissenberg number is different for the forward-directed flow and the backward-directed flow in the same microchannel. Ji et al. [2] numerically study the electroosmotic flow (EOF) of Oldroyd-B viscoelastic fluid through a 10:1 constriction microfluidic channel. Compared to EOF of Newtonian fluid, EOF of viscoelastic fluid becomes unstable when the PAA concentration and electric field exceed some critical values.

Joo’s group numerically and experimentally conducts novel studies on viscoelastic droplet motion. Ren et al. [3] numerically study migration of Oldroyd-B viscoelastic droplets on rigid surfaces with wettability gradients. The effects of parameters including droplet size, relaxation time, solvent viscosity, and polymer viscosity of the liquid on the migration speed and distance are investigated. Bai et al. [4] also investigate Janus droplet formation in a double Y-type microfluidic device filled with a power law shear-thinning fluid. Compared with Newtonian fluid, the Janus droplet is more readily generated in shear-thinning fluid. In the experimental study on the dynamics of liquid droplets under an electric field, Wei et al. [5] observe that viscoelastic droplets differ from Newtonian droplets, and further discuss the effects of viscoelasticity, the wettability, and the droplet size.

In terms of heat transfer for non-Newtonian fluids, Deng et al. [6] present transient hydrodynamical features and corresponding heat transfer characteristics in two-layer electroosmotic flow of power-law nanofluids in a microchannel. The results show that increase in nanoparticle volume fraction promotes heat transfer performance, and shear thickening feature of conducting nanofluid tends to suppress the effects of viscous dissipation and electrokinetic width on heat transfer. Hou et al. [7] numerically study heat and mass transport in a pseudo-plastic fluid past over a stretched porous surface in the presence of the Soret and Dufour effects. The effects of the tri-hybrid nanoparticles, the Dufour number, the heat generation parameter, the Eckert number, the buoyancy force parameters, and the porosity parameters on fluid temperature are reported. Hou et al. [8] also investigate the thermal transport of hydro-magnetized Carreau–Yasuda liquid passing over a permeable stretched surface, considering several important effects, including Joule heating, viscous dissipation, and heat generation/absorption. Sohail et al. [9] further report heat and mass transfer in three-dimensional second grade non-Newtonian fluid in the presence of a variable magnetic field. The maximum heat energy and improvement in motion of fluid particles are achieved using higher values of second grade fluid number. Parveen et al. [10] conduct heat transfer and entropy generation analysis in MHD axisymmetric flow of hybrid nanofluid. Detailed rheological impacts of involved parameters on flow variables and entropy generation number are demonstrated. Zhou et al. [11] provide a mathematical model for non-Newtonian Maxwell nanofluid flow with heat transmission over a porous spinning disc. The effects of some mathematical parameters on velocity, energy, concentration, and magnetic power are discussed. Rojas-Altamirano et al. [12] calculate the effective thermal conductivity of human skin using the Fractal Monte Carlo Method. In the study, tissue is described as a porous medium, and blood is considered a Newtonian and non-Newtonian fluid for comparative and analytical purposes.

Particle focusing in non-Newtonian fluids is also of great interest. Feng et al. [13] investigate particle focusing and separation in viscoelastic flow in a spiral channel. They explain the particle focusing position by the effects of inertial flow, viscoelastic flow, and Dean flow, and show that particle separation resolution can be improved in viscoelastic flow.

For the mixing in non-Newtonian fluids, Mei et al. [14] investigate electroosmotic micromixing of power law non-Newtonian fluid in a microchannel with wall-mounted obstacles and surface potential heterogeneity. Significant improvement in the mixing efficiency is achieved by increasing the obstacle surface zeta potential, the flow behavior index, the obstacle height, and the EDL thickness.

We would like to thank all the authors for submitting their papers to this Special Issue. We also thank all the reviewers for dedicating their time and helping to improve the quality of the submitted papers.
